# Multiple variation of right renal and gonadal vascularization: report of two cases

**DOI:** 10.1590/1677-5449.202300442

**Published:** 2024-03-25

**Authors:** Marcelo Calil Burihan, Silvio Antonio Garbelotti, Paulo Laino Cândido, Rodrigo Barbosa de Souza, Osvaldo Pelozo, Aluisio Andrade, Marco Antonio De Angelis

**Affiliations:** 1 Hospital Santa Marcelina – HSM, São Paulo, SP, Brasil.; 2 Faculdade Santa Marcelina – FASM, São Paulo, SP, Brasil.

**Keywords:** renal vein, accessory renal artery, gonadal vein, anatomical variation, veia renal, artéria renal acessória, veia gonadal, variação anatômica

## Abstract

We present two cases of multiple anatomical variations of the renal and gonadal vessels. The first case presented duplication of the renal vein and the presence of an accessory renal artery. However, the most interesting fact, in this case, was that the right gonadal vein emptied into the inferior right renal vein instead of ending in the inferior vena cava as would typically be the case. In the second case, we also found an accessory renal artery and the right gonadal vein emptied at the exact junction between the right renal vein and the inferior vena cava. Clinicians and surgeons should be familiar with anatomical variations to provide an accurate diagnosis during preoperative studies and to avoid surprises in abdominal surgical procedures.

## Resumo

Este estudo apresenta dois casos de variação anatômica múltipla de vasos renais e gonadais. O primeiro caso apresentou uma duplicação da veia renal e a presença de uma artéria renal acessória. Porém, o fato mais interessante nesse caso foi a veia gonadal direita desembocar na veia renal direita inferior em vez de terminar na veia cava inferior, como seria o normal. No segundo caso, além de também encontrarmos uma artéria renal acessória, a veia gonadal direita desembocava no exato ponto de junção entre a veia renal direita e a veia cava inferior. Clínicos e cirurgiões devem estar familiarizados com a presença de possíveis variações dos vasos renais e gonadais, sendo um conhecimento imprescindível para obter um diagnóstico mais preciso e para evitar surpresas em procedimentos cirúrgicos abdominais.

## INTRODUCTION

Anatomical variations in the vascular supply to the kidneys and gonads are not necessarily uncommon, yet authors are unanimous in emphasizing the importance of knowing about this type of vascular variation, as they are often found in retroperitoneal surgery, partial nephrectomy, transplantation, and angiographic procedures.^1^-^5^

Usually, the large renal veins are located anterior to the renal arteries and open into the inferior vena cava at almost a right angle. The right renal vein lies posterior to the descending part of the duodenum, is often less than 1 cm long, and because it is very short, makes safe nephrectomy difficult and may make resection of a cuff of the inferior vena cava necessary. The left renal vein is three times as long and the left is therefore the preferred side for living donor nephrectomy.^5^,^6^

In adults, the right gonadal vein enters the inferior vena cava directly on its anterolateral aspect at an acute angle about 2 cm below the left renal vein and occasionally it drains into the right renal vein,^7^ ending at a right angle as the left gonadal vein does, in which case, the likelihood of development of venous incompetence is increased.^2^,^6^

As for the arterial supply to the kidneys, we can consider a single renal artery to each kidney to be the standard pattern, usually present in almost 70% of individuals. Near the hilum of the kidney, each artery divides into an anterior and a posterior branch, which divide into segmental arteries that supply the renal vascular segments. Cases of anatomical variation represent about 30% and variations may occur in the level of origin, caliber, obliquity, and relationships with other structures.^5^-^8^

This study presents two cases of anatomical variation of the renal and gonadal vessels that are not only of interest to academics but are also of importance in daily practice, as much for correct interpretation of radiological exams as for local surgical interventions.

## CASE DESCRIPTIONS

During routine dissection of the abdominal region, vascular variations were found in supply to the right kidney in two adult human cadavers. These case reports were prepared at the Laboratory of Human Anatomy of the Santa Marcelina Medical School and were approved by the Ethics Committee, process nº 6.147.298/2023.

### Case 1

**Figure 1:**
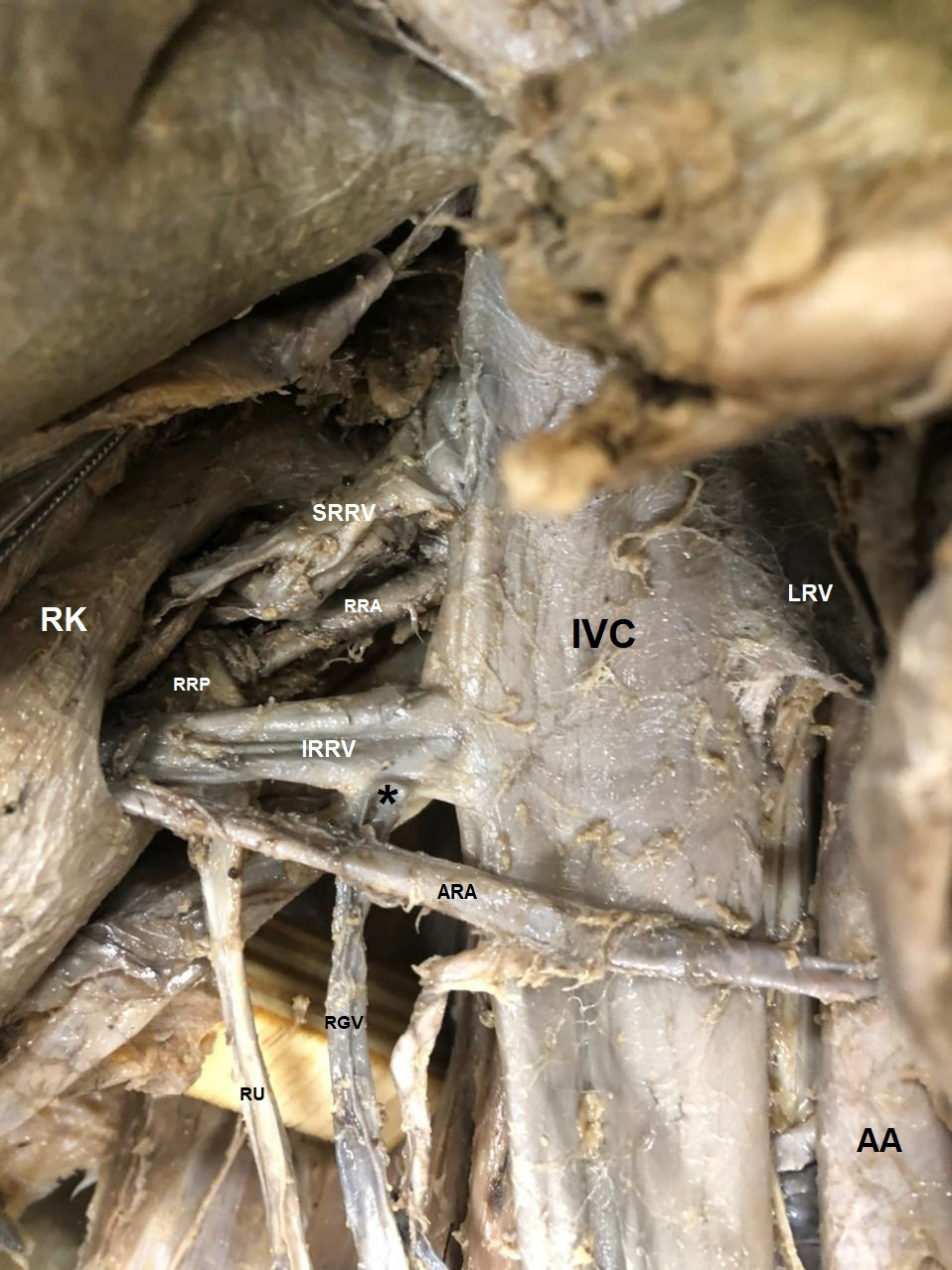
Anterior view of the right kidney and its blood vessels from Case 1. RK = Right kidney; IVC = Inferior vena cava; AA = abdominal aorta; LRV = Left renal vein; SRRV = Superior right renal vein (duplication); IRRV = Inferior right renal vein (duplication); RRA = Right renal artery; ARA = Accessory renal artery; RRP = Right renal pelvis; RU = Right ureter; RGV = Right gonadal vein; (*) point where the right gonadal vein empties into the inferior right renal vein.

A triple atypical variation of right kidney vascularization was found in a 37-year-old male cadaver. During access to the right renal lodge, we observed duplication of the renal vein in two smaller caliber veins that emerged from the real hilum of the kidney and drained directly into the inferior vena cava. They were both anterior to the renal pelvis and the right renal artery, one being superior and the other inferior to the renal artery. The right inferior renal vein opened into the vena cava at the same level as the left renal vein, while the superior renal vein opened at a point slightly above the left renal vein. Moreover, we observed that the right gonadal vein, which usually ends directly into the inferior vena cava, instead flowed into the inferior right renal vein near its entry point in the inferior vena cava. In addition to these venous variations, an accessory renal artery was found emerging from the anterior aspect of the abdominal aorta just above the origin of the inferior mesenteric artery, crossing anteriorly to the inferior vena cava, and entering the right hilum of the kidney anteriorly and inferiorly to the inferior renal vein and renal pelvis (Figure 1).

### Case 2

**Figure 2:**
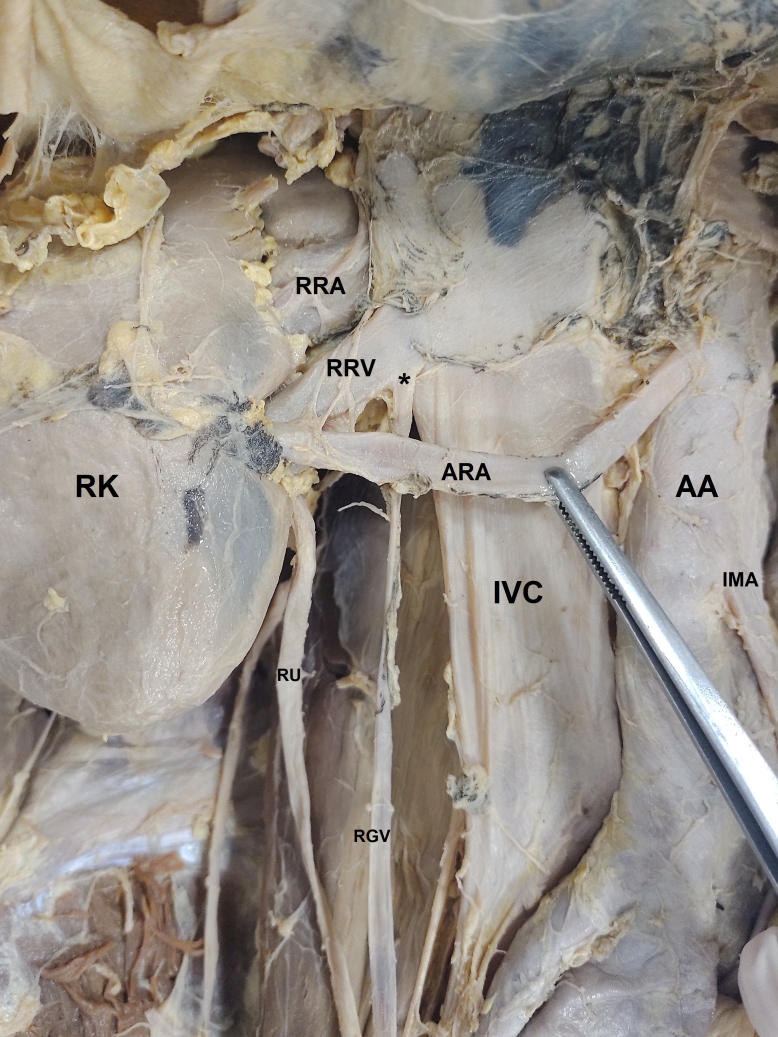
Anterior view of the right kidney and its blood vessels from Case 2. RK = Right kidney; IVC = Inferior vena cava; AA = abdominal aorta; RRV = Right renal vein; RRA = Right renal artery; ARA = Accessory renal artery; IMA = Inferior mesenteric artery; RU = Right ureter; RGV = Right gonadal vein; (*) point where the right gonadal vein empties at the junction of the right renal vein and the inferior vena cava.

In this case, we observed a vascular variation in a 57-year-old male cadaver. The right gonadal vein opened inferiorly at the exact point of junction of the right renal vein with the inferior vena cava. The renal vein was not duplicated. We also observed an accessory renal artery emerging from the anterior aspect of the abdominal aorta slightly above the origin of the inferior mesenteric artery, crossing anterior of the inferior vena cava, and entering the hilum of the kidney anteriorly and inferiorly to the right renal vein and renal pelvis (Figure 2).

There were no variations in the vessels leading to the left kidney in either case or any other vascular abnormalities found during dissection of the abdominal cavities.

## DISCUSSION

Anatomical variations of the renal arteries are not uncommon and occur in up to 30% of cases.^5^,^8^-^12^ Accessory arteries are the most common and account for a third of these cases, generally arising from the aorta below the main renal artery.^5^,^8^ Accessory renal arteries are differentiated into hilar and polar arteries. Hilar arteries differ from the others because they enter the kidney through the renal hilum and then branch into segmental arteries, sharing responsibility for supplying the kidney with the main renal artery, whereas polar arteries penetrate the kidney either through the upper or lower pole, limiting their contribution to this specific region.^8^-^12^

We present two cases in which an accessory renal artery was observed. Such variations can be explained by the embryological development of kidneys and gonads from the intermediate mesoderm of the mesonephric crest and vascularization of both organs from the lateral mesonephric branches of the dorsal aorta.^13^,^14^

Accessory arteries do not constitute duplication of vessels, but rather one or more segmental arteries (terminal branches) are each exclusively responsible for a portion of the kidney.^4^,^6^ Therefore, if an accessory artery is damaged, the part of the kidney supplied by it will likely become ischemic.^1^,^13^

Use of kidneys from living donors with multiple renal arteries has been discouraged due to the increased risk to the donor of obtaining a mutual aortic cuff, the technical difficulty of completing multiple arterial anastomoses, prolonged ischemia time, and poorly controlled hypertension from segmental allograft alterations.^1^ In addition to the possibility of nutcracker syndrome, in which compression of the left renal vein is caused by the superior mesenteric artery.^4^

As with arteries, the causes of variation in the origin, course, and branches of the renal and gonadal veins can be attributed to embryological origin.^2^,^14^ Development of the renal and gonadal veins is often closely related to the inferior vena cava. Embryogenesis of these veins involves the development, regression, anastomosis, and replacement of three pairs of venous channels: the posterior cardinal, subcardinal, and supracardinal veins. Anastomosis between the supracardinal and subcardinal veins, which occurs bilaterally, forms the renal segment of the inferior vena cava. The gonadal vein develops from the caudal part of the subcardinal vein and drains into the suprasubcardinal anastomosis. On the right side, this suprasubcardinal anastomosis and a small portion of the sub-cardinal vein are incorporated into the formation of the inferior vena cava. Thence, the right gonadal vein usually drains into the inferior vena cava.^2^,^15^,^16^

In the two cases presented here, there was only variation in the venous drainage pattern on the right side, a variation that appears to be less common. According to a study presented by Baptista-Silva et al.^8^ that analyzed 342 cases of variation in the renal vein, only 6.4% of the cases occurred on the right side, about a third of which had a duplicated renal vein with just a single case of tributary gonadal vein. The authors reported that the variation in syntopy of a single vein is much more common (61.29%), while three or more renal veins are found less commonly (9.7%). Cicek et al.^5^ found slightly higher rates of multiple right-sided renal veins, at 28.2%, in a retrospective study of 1,859 voluntary donors during an 11-year follow-up.

Furthermore, variation in the venous drainage pattern of the gonadal veins was noted in both cases, but with different patterns. The differences in regular testicular venous drainage patterns probably explain the fact that 95% of varicoceles occur on the left side, where the gonadal vein is a few centimeters higher than the right and performs two bends at two right angles to flow into the inferior vena cava. On the other hand, the right testicular vein ends directly in the inferior vena cava at an acute angle, indicating higher hemodynamic pressure in the left testicular vein than in the right.^17^

In the first case, we noted that the gonadal vein opened directly into the right renal vein at a 90° angle. This variation was also described in a case report by Nayak.^18^ Ahlberg et al.^19^ observed this type of variation in only 8.3% of cases in 84 autopsies performed and Asala et al.^20^ observed it only twice (1.3%) in 150 cadavers studied. If we extrapolate the knowledge of the hemodynamic resistance that occurs on the left side, this type of variation may result in right-sided or bilateral varicocele, as documented by Zini et al.^21^

In the second case, the gonadal vein ended obliquely at the point of junction of the renal vein with the inferior vena cava; we did not find similar reports in the literature. In this case, we can only assume that the oblique termination of the vein, although unusual, does not seem to offer the same hemodynamic resistance described in the previous case, since the retrograde flow through this vein tends to be lower due to its position about the inferior vena cava.

The origin and course of the vessels to the kidneys must be carefully identified to preserve normal blood flow and to prevent possible anatomical variations from becoming clinically complex abnormalities.^3^,^4^ Radiologists, vascular surgeons, urologists, and oncologists should be familiar with anatomical variations to provide an accurate diagnosis during preoperative studies.
